# Supplementary material for: From sequence to enzyme mechanism using multi-label machine learning
Source: BMC Bioinformatics. 2014 May 19;15:150. doi: 10.1186/1471-2105-15-150 (PMC4229970; doi:10.1186/1471-2105-15-150)
Supplement: Additional file 2 — Java code of ml2db. Additional file ml2db_code.tar.gz contains the Java source code to run the multi-label machine learning experiments and save the results to database. The code’s Javadoc is included. [file 1471-2105-15-150-S2.zip › additional file 2/ml2db/ml2db/doc/main/ml2db/al/ActiveLearning.html]

ActiveLearning


JavaScript is disabled on your browser.


- Overview
- Package
- Class
- Use
- Tree
- Deprecated
- Index
- Help

- Prev Class
- Next Class

- Frames
- No Frames

- All Classes

- Summary:
- Nested |
- Field |
- Constr |
- Method

- Detail:
- Field |
- Constr |
- Method


main.ml2db.al

## Class ActiveLearning

- java.lang.Object
- - uk.ac.standrews.utils.main.Initialised
  - - main.ml2db.al.ActiveLearning

- All Implemented Interfaces:
  :   uk.ac.standrews.utils.main.Initialisable

  ---

    

  ```
  public class ActiveLearning
  extends uk.ac.standrews.utils.main.Initialised
  ```

  Executes pool-based active (machine) learning for nearest neighbours.
  Approximate pseudo-code: (1) input: a set of unlabelled instances (U) and a
  set of labelled instances L (which can be empty) (2) calculate an
  utility/desirability metric for each unlabelled instance (can be random,
  based on confidence, based on distance to the other instances or other
  method) (4) pick the most useful/desirable instance (5) label the instance
  (that is, add it to the set of labelled instances L ) (6) go to (2) until at
  least k+1 instances have been labelled (where k is the desired number of
  neighbours) (7) train on L and test on U (8) record evaluation metrics (9) go
  to (2) until no more unlabelled instances are available

  Version:
  :   7 Jul 2011

  Author:
  :   Luna De Ferrari ldf(at)st-andrews.ac.uk

- - ### Field Summary

    Fields

    | Modifier and Type | Field and Description |
    | --- | --- |
    | `static java.lang.String` | `EUCLIDEAN_DISTANCE_NAME` |

    - ### Fields inherited from class uk.ac.standrews.utils.main.Initialised

      `m_initialisationIsCorrect`
  - ### Constructor Summary

    Constructors

    Constructor and Description

    `ActiveLearning(ExperimentDetails experimentData,
    int numberOfNeighbours,
    java.lang.String resultsDirectoryPath)`

    `ActiveLearning(ExperimentDetails experimentData,
    int numberOfNeighbours,
    java.lang.String resultsDirectoryPath,
    int stepLimit)`
  - ### Method Summary

    Methods

    | Modifier and Type | Method and Description |
    | --- | --- |
    | `java.util.TreeMap<java.lang.String,java.lang.Double>` | `calculateUtilities(int stepId)` Calculate utility metric for each instance. |
    | `AlDataSaver` | `getDataSaver()` |
    | `java.util.Vector<uk.ac.standrews.utils.main.database.TableRow>` | `getEvaluationResultsRows()` |
    | `ExperimentDetails` | `getExperimentDetails()` |
    | `int` | `getExperimentId()` |
    | `java.util.Vector<java.lang.String>` | `getInstancesNames()` |
    | `IdMultiLabelInstances` | `getLabelled()` |
    | `IdMultiLabelInstances` | `getLabInstances()` |
    | `java.io.File` | `getLogFile()` |
    | `int` | `getNumberOfNeighbours()` |
    | `java.util.Calendar` | `getStartTime()` |
    | `int` | `getStepId()` |
    | `int` | `getStepLimit()` |
    | `MulanLearner` | `getTrainedLearner()` |
    | `IdMultiLabelInstances` | `getUnlabelled()` |
    | `boolean` | `hasEnoughLabelledInstances()` |
    | `void` | `initialise(ExperimentDetails experimentManager, int numberOfNeighbours, java.lang.String resultsDirectoryPath, int stepLimit)` |
    | `boolean` | `isGenerateGraphViz()` |
    | `boolean` | `isTest()` |
    | `static void` | `main(java.lang.String[] args)` |
    | `java.lang.String[]` | `run()` |
    | `java.lang.Object[]` | `runActiveLearningStep(int stepId)` Calculate utility metric for each instance, pick best (query instance), add query instance to labelled set and delete from unlabelled set. |
    | `void` | `setDebug(boolean debug)` |
    | `void` | `setDebugEvaluationResults(boolean debugEvaluationResults)` |
    | `void` | `setEnoughLabelledInstances()` |
    | `void` | `setLabInstances(IdMultiLabelInstances labInstances)` |
    | `void` | `setLogFile(java.io.File logFile)` |
    | `void` | `setTest(boolean test)` |
    | `void` | `setTrainedLearner(MulanLearner mulanLearner)` |
    | `void` | `setUnlabInstances(IdMultiLabelInstances unlabInstances)` |

    - ### Methods inherited from class uk.ac.standrews.utils.main.Initialised

      `initialisationIsCorrect, setInitialisation, setInitialisationIsCorrect, setInitialisationNotCorrect`
    - ### Methods inherited from class java.lang.Object

      `equals, getClass, hashCode, notify, notifyAll, toString, wait, wait, wait`

- - ### Field Detail


    - #### EUCLIDEAN\_DISTANCE\_NAME

      ```
      public static final java.lang.String EUCLIDEAN_DISTANCE_NAME
      ```

      See Also:
      :   Constant Field Values
  - ### Constructor Detail


    - #### ActiveLearning

      ```
      public ActiveLearning(ExperimentDetails experimentData,
                    int numberOfNeighbours,
                    java.lang.String resultsDirectoryPath)
      ```

      Parameters:
      :   `experimentData` -
      :   `numberOfNeighbours` -
      :   `resultsDirectoryPath` -
      :   `stepLimit` - the number of steps the simulation should run for


    - #### ActiveLearning

      ```
      public ActiveLearning(ExperimentDetails experimentData,
                    int numberOfNeighbours,
                    java.lang.String resultsDirectoryPath,
                    int stepLimit)
      ```

      Parameters:
      :   `experimentData` -
      :   `numberOfNeighbours` -
      :   `resultsDirectoryPath` -
      :   `stepLimit` - the number of steps the simulation should run for
  - ### Method Detail


    - #### calculateUtilities

      ```
      public java.util.TreeMap<java.lang.String,java.lang.Double> calculateUtilities(int stepId)
      ```

      Calculate utility metric for each instance.

      Returns:
      :   the utility metric for each instance name.


    - #### getDataSaver

      ```
      public AlDataSaver getDataSaver()
      ```


    - #### getEvaluationResultsRows

      ```
      public java.util.Vector<uk.ac.standrews.utils.main.database.TableRow> getEvaluationResultsRows()
      ```


    - #### getExperimentDetails

      ```
      public ExperimentDetails getExperimentDetails()
      ```


    - #### getExperimentId

      ```
      public int getExperimentId()
      ```


    - #### getInstancesNames

      ```
      public java.util.Vector<java.lang.String> getInstancesNames()
      ```


    - #### getLabelled

      ```
      public IdMultiLabelInstances getLabelled()
      ```


    - #### getLabInstances

      ```
      public IdMultiLabelInstances getLabInstances()
      ```


    - #### getLogFile

      ```
      public java.io.File getLogFile()
      ```


    - #### getNumberOfNeighbours

      ```
      public int getNumberOfNeighbours()
      ```


    - #### getStartTime

      ```
      public java.util.Calendar getStartTime()
      ```


    - #### getStepId

      ```
      public int getStepId()
      ```


    - #### getStepLimit

      ```
      public int getStepLimit()
      ```

      Returns:
      :   m\_stepLimit


    - #### getTrainedLearner

      ```
      public MulanLearner getTrainedLearner()
      ```


    - #### getUnlabelled

      ```
      public IdMultiLabelInstances getUnlabelled()
      ```


    - #### hasEnoughLabelledInstances

      ```
      public boolean hasEnoughLabelledInstances()
      ```


    - #### initialise

      ```
      public void initialise(ExperimentDetails experimentManager,
                    int numberOfNeighbours,
                    java.lang.String resultsDirectoryPath,
                    int stepLimit)
      ```


    - #### isGenerateGraphViz

      ```
      public boolean isGenerateGraphViz()
      ```

      Returns:
      :   m\_generateGraphViz


    - #### isTest

      ```
      public boolean isTest()
      ```

      Returns:
      :   m\_test


    - #### run

      ```
      public java.lang.String[] run()
      ```


    - #### runActiveLearningStep

      ```
      public java.lang.Object[] runActiveLearningStep(int stepId)
      ```

      Calculate utility metric for each instance, pick best (query instance),
      add query instance to labelled set and delete from unlabelled set.

      Parameters:
      :   `trainedLearner` -

      Returns:
      :   the query (most useful) instance


    - #### setDebug

      ```
      public void setDebug(boolean debug)
      ```


    - #### setDebugEvaluationResults

      ```
      public void setDebugEvaluationResults(boolean debugEvaluationResults)
      ```

      Parameters:
      :   `debugEvaluationResults` - m\_debugEvaluationResults to set


    - #### setEnoughLabelledInstances

      ```
      public void setEnoughLabelledInstances()
      ```


    - #### setLabInstances

      ```
      public void setLabInstances(IdMultiLabelInstances labInstances)
      ```

      Parameters:
      :   `labInstances` - m\_labInstances to set


    - #### setLogFile

      ```
      public void setLogFile(java.io.File logFile)
      ```


    - #### setTest

      ```
      public void setTest(boolean test)
      ```

      Parameters:
      :   `test` - m\_test to set


    - #### setTrainedLearner

      ```
      public void setTrainedLearner(MulanLearner mulanLearner)
      ```


    - #### setUnlabInstances

      ```
      public void setUnlabInstances(IdMultiLabelInstances unlabInstances)
      ```

      Parameters:
      :   `unlabInstances` - m\_unlabInstances to set


    - #### main

      ```
      public static void main(java.lang.String[] args)
      ```


- Overview
- Package
- Class
- Use
- Tree
- Deprecated
- Index
- Help

- Prev Class
- Next Class

- Frames
- No Frames

- All Classes

- Summary:
- Nested |
- Field |
- Constr |
- Method

- Detail:
- Field |
- Constr |
- Method
